# Age and site-specific pattern on encrustation of charophytes

**DOI:** 10.1186/s40529-018-0247-5

**Published:** 2018-12-19

**Authors:** Anne Herbst, Hendrik Schubert

**Affiliations:** University of Rostock, Biosciences, Albert-Einstein-Straße 3, 18059 Rostock, Germany

**Keywords:** Carbonate precipitation, Age gradient, Plant thallus, Photosynthesis, Potassium

## Abstract

Encrustation and element content (Ca, Fe, K, Mg and P) of charophytes was studied along plant thalli to investigate the dependency of thallus age and site-specificity. Charophytes were collected from five sampling sites (Angersdorfer Teiche, Asche, Bruchwiesen, Krüselinsee and Lützlower See) which were distinct with respect to water chemistry. Furthermore, photosynthesis was measured to identify the physiological state of plants in habitat waters and with the addition of different ion concentrations (Ca^2+^, K^+^, Mg^2+^ and Na^+^). Age pattern on encrustation of charophytes was site-specific: carbonate content increased from the youngest to the oldest part (Angersdorfer Teiche), younger parts were less encrusted than older parts in Asche, Bruchwiesen and Krüselinsee, whereas encrustation in Lützlower See was the same along plants thallus. Charophytes showed species-specific encrustation in investigated sites. Encrustation of *C. hispida* in Angersdorfer Teiche was also as high as of individuals from hard-water lakes irrespective of 10.15 mS cm^−1^ (salinity of 6.3). For species growing in Angersdorfer Teiche, K/Na content and photosynthesis was lowest when compared to other sites. Photosynthesis of charophytes was enhanced after the addition of KCl and adversely affected by CaCl_2_, MgCl_2_ and NaCl. In summary, it was shown that encrustation of charophytes in water sites with strong ion anomalies could be as high as in hard-water lakes. It is assumed that ion composition, rather than ion concentration of Na^+^, Mg^2+^ and SO_4_^2−^, impact on the encrustation of charophytes. The age pattern on encrustation in this study showed a strong site-specificity, whereas encrustation of charophytes was species-specific. Ion concentrations, either of habitats or actively added in laboratory measurements, impact on encrustation, element content and photosynthesis of charophytes.

## Background

Charophytes are macrophytes of the order Charales Lindley 1836 belonging to the streptophyta (Jeffrey 1967; Stewart and Mattox 1975). This lineage also includes the embryophyta, thus charophytes are closely related to land plants (Jeffrey 1967; McCourt 1995; Nishiyama et al. 2018). Charophytes can form dense meadows which provide habitats and shelter for epiphytes, invertebrates and fish, indicating their important ecological function in the ecosystem (Kairesalo et al. 1987; Kingsford and Porter 1994; Kuczyńska-Kippen 2007). The presence or absence of charophytes seems to depend on abiotic lake characteristics; water chemistry especially plays an important role (van den Berg et al. 1998a; Blindow et al. 2014). They often occur in waters with low nutrient concentrations. Therefore, some charophytes can serve as bioindicators for nutrient-poor conditions (Krause 1981; Doege et al. 2016). In clear-water ecosystems charophytes are more competitive than other macrophytes but are outcompeted under eutrophic conditions (Ozimek and Kowalczewski 1984; Pieczyńska et al. 1988; Blindow 1992). However, charophytes are not limited to oligotrophic waters, also inhabiting eutrophic waters when niches are available (van den Berg et al. 1998b; Schubert et al. 2018). In hard-water lakes, charophytes encrust on the plant surface (Wahlstedt 1875; Sand-Jensen et al. 2018). Calcium carbonate content can thereby account for up to 80% of plant dry weight (Pukacz et al. 2016a). In the case of heavy encrustation of charophytes, it has been described that supersaturation with calcium carbonate of the water was not required (Nõges et al. 2003; Kufel et al. 2016). Several authors pointed out the importance of site-specificity, and the influence that depth, temperature, pH, and water chemistry parameters have on the encrustation of charophytes (Kufel et al. 2013, 2016; Pukacz et al. 2016a, b). So far age dependent encrustation was analyzed by Kawahata et al. (2013); younger internodes of *C. globularis* Thuill. 1799 were less encrusted than older ones. In situ studies on encrustation regarding age and site-specificity have never been done. Therefore, encrustation was analyzed in parts of corticated thallus for *C. canescens* Loisel. 1810, *C. hispida* L. 1753, *C. subspinosa* Rupr. 1846 and *C. tomentosa* L. 1753 from five areas of water in Germany. We investigated whether carbonate and element (Ca, Fe, K, Mg and P) contents of plant dry weight (DW) are dependent on thallus age and sites sampled. Furthermore, photosynthesis was measured to identify the physiological state of plants in habitat waters and with the addition of different ion concentrations (Ca^2+^, K^+^, Mg^2+^ and Na^+^).

## Materials and methods

### Sampling sites

**Fig. 1:**
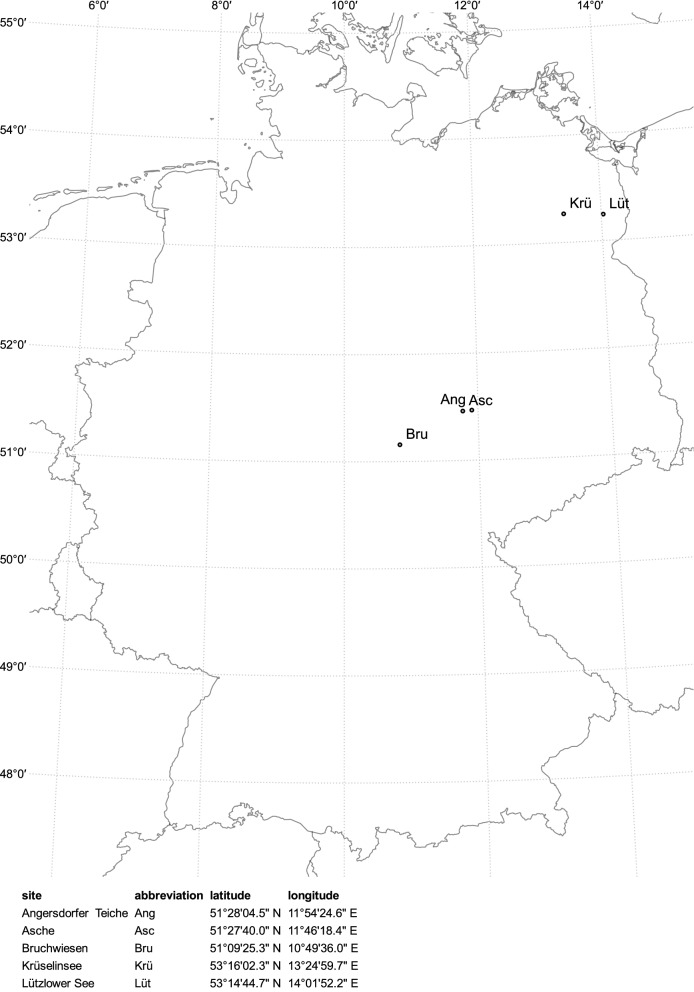
Map of investigated sampling sites (QGIS version 3.2, GeoBasis-DE/BKG 2018). Sites are abbreviated as first three letters: Krüselinsee (Krü), Lützlower See (Lüt), Angersdorfer Teiche (Ang), Asche (Asc) and Bruchwiesen (Bru)

In July 2016 five areas of water were investigated in the northeast and eastern part of Germany (Fig. 1). These included two residual mining holes: Angersdorfer Teiche, a clay pit filled with rain and groundwater, and Asche in Teutschenthal, which is located close to a potassium mining heap, in the lake area Mansfelder region. Bruchwiesen, a natural spring, in Bad Tennstedt was also sampled, as well as two hard-water lakes, Krüselinsee in Mecklenburg Lake District and Lützlower See in Uckermark.

### Sampling processing

**Fig. 2:**
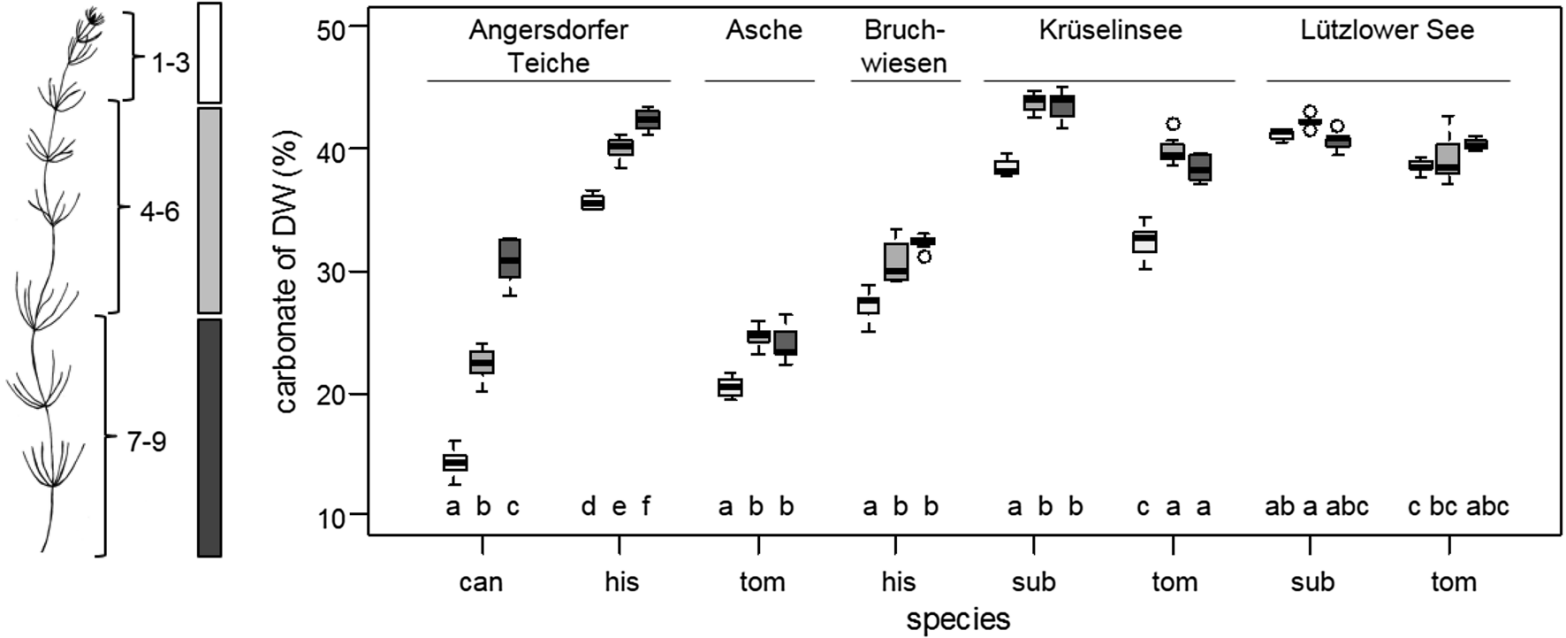
Comparison of carbonate content based on DW (%) of *C. canescens* (can), *C. hispida* (his), *C. subspinosa* (sub), and *C. tomentosa* (tom) from sampling sites (Angersdorfer Teiche, Asche, Bruchwiesen, Krüselinsee, and Lützlower See). Plants were analyzed in first to third (white), forth to sixth (light grey), seventh to ninth (dark grey boxplots) whorls and internodes. Box plots include whiskers (5–95% of variability) and outliers (points). Different letters indicate significant difference within the same sampling site (Tukey HSD post hoc test, p < 0.05)

Plant material was collected at a depth of 0.5–1.5 m from the shore, or by snorkeling. Undamaged plants of *C. canescens*, *C. hispida*, *C. subspinosa*, and *C. tomentosa* were picked. In the laboratory, epibionts were removed gently with a brush. For further analyses, plants were analyzed in parts of thalli. First to third, forth to sixth, seventh to ninth whorls and internodes were used for analysis (Fig. 2). Side branches and other parts of thalli were removed.

Carbonate proportion of plant dry weight was determined by the loss of ignition (LOI) method as described by Heiri et al. 2001. Sample size was n = 7 for the LOI analysis. The samples were dried at 60 °C (UM 400, Memmert) overnight. Plant DW was weighed and combusted in crucibles in a two-step process, at 550 °C (DW_550_) and 925 °C (DW_925_) for two hours, respectively (LE 6/11/B 150, Nabertherm). Carbonate content of DW (%) was calculated of the carbonate bounded CO_2_ loss multiplied by 1.36 (fraction of CO_2_ in CO_3_^2−^) after Pełechaty et al. 2013 (Eq. 1).

1 $${\text{carbonate content of DW }}\left( {\text{\%}} \right) = \left( {\frac{{{\text{DW}}550 - {\text{DW}}925}}{{{\text{DW}}925}} \times 100} \right) \times 1.36$$

Element contents of Ca, Fe, K, Mg and P were measured with inductively coupled plasma-optic emission spectroscopy (ICP-OES). Plant DW was ground and 0.1 g was weighted for the digestion. Samples were extracted in duplicate with 5 ml HNO_3_ (65%) and 3 ml H_2_O_2_ (30%) for 1.5 h in CEM MARS 6 microwave. After extraction, samples were filled up to 25 ml with ultrapure water and were filtered (601P, Rotilabo). Samples were analyzed spectrometrically for Ca (317.9 nm), Fe (238.2 nm), K (766.5 nm), Mg (285.2 nm) and P (214.9 nm) with Optima 8300 (Perkin Elmer, Singapore).

Photosynthesis of *C. canescens*, *C. hispida* from Angersdorfer Teiche, *C. hispida* from Asche, and *C. tomentosa* from Bruchwiesen was measured by means of O_2_ evolution with a Clark-type electrode (Microelectrodes, USA). Within a day after sampling, plants were dark adapted at least for 30 min and were set in a temperature-controlled 2.5 ml cuvette (15 °C). The cuvette was filled with habitat medium which was stirred during measurement. Photon flux densities were applied by a light dispenser system (MK2, Illuminova, Sweden) described by Wolfstein and Hartig (1998). Dark respiration was measured for 10 min, followed by increasing irradiation levels of 14, 18, 31, 102, 494, 912, 1462, 1811, 2120 µmol photons m^−2^ s^−1^. Each irradiation level was set for 3.3 min; measurement took 40 min in total.

Photosynthesis of *C. tomentosa* from Asche was measured with the addition of CaCl_2_, KCl, MgCl_2_ and NaCl. Plants were cultivated in habitat water and at a constant temperature (15 °C) and light (50–80 µmol photons m^−2^ s^−1^ on a 12 h/12 h light–dark cycle) to allow consistent conditions for measurements. Light saturation irradiance (I_s_) of cultivated *C. tomentosa* was calculated in preliminary measurements (I_s_ = 120 µmol m^−2^ s^−1^). For 20 min measurements, 1.5 × I_s_ (1.5I_s_ = 180 µmol m^−2^ s^−1^) was used to gain light saturation. After a dark period of 3 min, constant irradiance level of 180 µmol m^−2^ s^−1^ was set. Concentration of 1.2 mmol L^−1^ of chloride salt was added at minutes 5, 7, 9, 11, 13, 15, and 17 of measurement. The end concentration was 8.4 mmol L^−1^ added chloride salt in the cuvette. Control measurements were conducted without addition of chloride salt.

Photosynthesis/irradiance (P/I) curve was calculated using the model of Walsby (1997) to determine photosynthesis (P), maximum photosynthesis (P_m_), dark respiration (R_d_), *α* (slope of P/I curve at limiting irradiance), light compensation point (I_c_ = R_d_/*α*), and light saturation irradiance (I_s_ = P_m_/*α*). Photosynthetic parameters were based on chlorophyll *a* (chl *a*). Pigment content of first to third whorls and internodes were extracted with 3 ml of *N*,*N*-dimethylformamide. Samples were incubated overnight at 4 °C. Extinction at 470, 630, 647, 664, and 750 nm was measured using a Lambda 2 spectrometer (Perkin Elmer, Germany). Chl *a* content was calculated after Wellburn (1994).

Water samples were taken in 50 ml tubes above plant meadows, closely beneath the water surface. pH value and conductivity were measured directly at sampling sites with HACH HQ40d (Hach-Lange, Germany). The water samples were analyzed at Helmholtz Centre for Environmental Research (UFZ)—Central Laboratory for Water Analytics and Chemometrics. The concentrations of Ca^2+^, Cl^−^, K^+^, Mg^2+^, Na^+^, and SO_4_^2−^ were measured with an ion chromatograph (Dionex ICS 3000, Thermo Fisher, USA). Total inorganic carbon (TIC) was determined with a Dimatoc 2000 (DIMATEC, Germany). Water samples were measured in accordance with EN ISO 14911:1999 and EN ISO 10304:2009-1.

### Statistical analyses

Data were analyzed for normality and homogeneity. In cases of normal and homogeneous distribution, data were analyzed with an analysis of variance (ANOVA) and post hoc Tukey HSD test. For no normal distributed data, a non-parametric test (Kruskal–Wallis test and Fisher Least Significant Difference (LSD) post hoc test) with Bonferroni correction for p-values adjustment was performed (de Mendiburu 2016). A principal component analysis (PCA) based on standardized data of element contents of Ca, Fe, K, Mg and TP of plant DW was conducted (Oksanen et al. 2017). Significance levels were set to a p-value ≤ 0.05. All statistical analyses were performed using R (R Core Team 2017).

## Results

### Water chemistry

Water chemistry data of investigated sites are listed in Table 1. The sampling sites were distinct with respect to water chemistry, exhibiting enhanced ion concentrations in Angersdorfer Teiche, Asche, and Bruchwiesen. The highest ion concentrations of Ca^2+^, Cl^−^, K^+^, Na^+^ and SO_4_^2−^ were found in the gips pit Angersdorfer Teiche. Mg^2+^ was an exception and the highest concentration was obtained in Asche. Bruchwiesen had high Ca^2+^ and SO_4_^2−^ concentrations compared to Krüselinsee and Lützlower See. Both hard-water lakes had the lowest conductivity of investigated waters. Total inorganic carbon (TIC) concentration was highest in Bruchwiesen and lowest in Angersdorfer Teiche. pH was around 9 in Angersdorfer Teiche, Krüselinsee and Lützlower See, 7.9 in Asche, and lowest in Bruchwiesen with 7.4.

**Table 1:** Water chemistry data of sampling sites investigated and species sampled

Site	Species	pH	Cond.	Ca^2+^	Cl^−^	K^+^	Mg^2+^	Na^+^	SO_4_^2−^	TIC
Angersdorfer Teiche	can, his	9.1	10.15	660	2390	82.5	455	1560	2550	15.7
Asche	tom	7.9	5.03	431	472	33.7	462	626	2380	38.1
Bruchwiesen	his	7.4	1.39	276	41	3.0	51	23	577	64.1
Krüselinsee	sub, tom	9.3	0.26	29	18	2.4	6.2	9.3	29	22.2
Lützlower See	sub, tom	9.0	0.80	91	106	3.7	21	28	152	30.0

Sites and species are abbreviated as first three letters. Listed are pH, and conductivity (mS cm^−1^) and concentrations of Ca^2+^, Cl^−^, K^+^, Mg^2+^, Na^+^, SO_4_^2−^, and TIC (mg L^−1^)

### Encrustation

Encrustation was analyzed in parts of plant thalli (Fig. 2). A pattern of lowest carbonate content in the first to third, middle in the forth to sixth, and highest in the seventh to ninth whorls and internodes was found for both species (*C. canescens* and *C. hispida*) from Angersdorfer Teiche (Fisher LSD post hoc test, p < 0.01). In Asche, Bruchwiesen, and Krüselinsee, the younger parts of *C. hispida*, *C. subspinosa*, and *C. tomentosa* were less encrusted than the middle and older parts of the plant thalli (Fisher LSD post hoc test, p < 0.001). In Lützlower See, *C. subspinosa* and *C. tomentosa* exhibited no significant differences in encrustation between their respective parts of the thalli.

Furthermore, encrustation was species-specific for species in Angersdorfer Teiche. *C. hispida* encrusted higher than *C. canescens*. Site-specific encrustation was found for *C. hispida* growing in Angersdorfer Teiche and Bruchwiesen with a lower carbonate proportion of *C. hispida* in Bruchwiesen (Fisher LSD post hoc test, p < 0.01). Also *C. tomentosa* encrustation was site-specific in Asche compared to individuals from Krüselinsee and Lützlower See. Encrustation of *C. tomentosa* was lower in Asche than in Krüselinsee and Lützlower See (Fisher LSD post hoc test, p < 0.001).

### Element composition

**Fig. 3:**
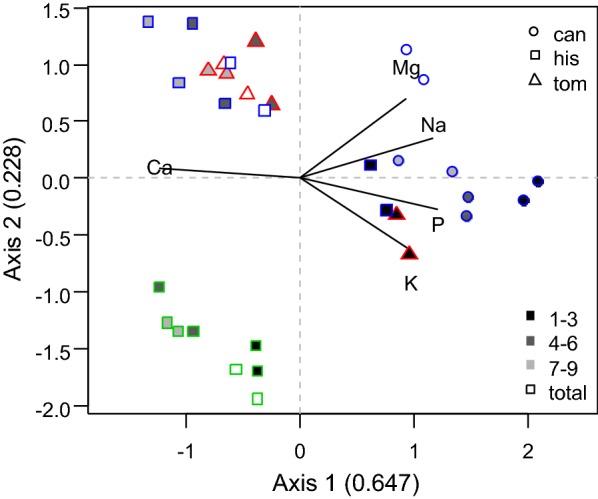
PCA plot of element contents (Ca, K, Mg, Na, and P) of charophytes from Angersdorfer Teiche (blue), Asche (red), and Bruchwiesen (green). Species were plotted with different symbols; background of symbols indicates parts of thallus, see legend

Element content based on plant dry weight was analyzed for species from habitats with high ion concentrations (Angersdorfer Teiche, Asche, and Bruchwiesen) to investigate the impact thereof (Fig. 3 and Table 2). In the PCA plot, *C. hispida* from Bruchwiesen was separated from the species of Angersdorfer Teiche and Asche. Habitat differences were pronounced by means of lowest contents of Mg and Na in *C. hispida* from Bruchwiesen (Table 2). Further, parts of the thallus were ordered along the axis 1 which was characterized most by Ca, P, and Na contents.

**Table 2:** Comparison of element contents (mean ± SD) of charophytes from Angersdorfer Teiche, Asche, and Bruchwiesen

Site	Species	Ca	K	Mg	Na	P	K/Na
Angersdorfer Teiche	can	94 ± 34.4 c	16.4 ± 1.7 a	12.6 ± 0.8 a	26.0 ± 2.3 a	1.6 ± 0.4 a	0.6 ± 0.1 d
	his	251.3 ± 41.8 a	11.2 ± 3.5 b	10.7 ± 0.7 b	14.7 ± 4.2 b	0.9 ± 0.4 b	0.8 ± 0.1 c
Asche	tom	158 ± 35.8 b	11.7 ± 3.6 b	11.7 ± 0.7 a	8.4 ± 2.3 c	0.9 ± 0.4 b	1.4 ± 0.1 b
Bruchwiesen	his	215.4 ± 17.3 a	14.7 ± 2.2 ab	6.3 ± 0.6 c	2.9 ± 1.0 d	1.0 ± 0.3 ab	5.5 ± 1.4 a

Ca, K, Na, Mg, and P contents are presented as g kg^−1^ DW; K/Na mass ratios are calculated from weight. Species are abbreviated: *C. canescens* (can), *C. hispida* (his), and *C. tomentosa* (tom). Sample size was n = 8 for the element analysis. Different letters indicate significant differences in element content (Tukey HSD post hoc test, p < 0.05)

Regarding the element content, Ca was lowest in *C. canescens* and highest in *C. hispida*, both from Angersdorfer Teiche. K content was significantly higher in *C. canescens* compared to *C. hispida* (Angersdorfer Teiche) and *C. tomentosa* (Asche). Na content was different for all species investigated; highest in *C. canescens* (Angersdorfer Teiche) and lowest in *C. hispida* (Bruchwiesen). The ratio of K/Na was low (< 1) for both species from Angersdorfer Teiche and highest in *C. hispida* from Bruchwiesen. P content was significantly higher in *C. canescens* compared to *C. hispida* (Angersdorfer Teiche) and *C. tomentosa* (Asche).

### Photosynthesis

Photosynthesis was measured in plants from Angersdorfer Teiche, Asche and Bruchwiesen (Table 3). P_m_ of *C. canescens* (33.8 ± 9.6 1 mmol O_2_ h^−1^g chl *a*^−1^) and *C. hispida* (42.1 ± 6.1 mmol O_2_ h^−1^ g chl *a*^−1^) from Angerdorfer Teiche were not significantly different to *C. tomentosa* from Asche (64.3 ± 20.2 mmol O_2_ h^−1^ g chl *a*^−1^) and *C. hispida* from Bruchwiesen (64.7 ± 18.8 mmol O_2_ h^−1^ g chl *a*^−1^). α, R_d_ and I_s_ were also similar for species measured. Significant difference was found for the light compensation point; *C. canescens* from Angersdorfer Teiche had significantly lower I_c_ than *C. hispida* from Bruchwiesen.

**Table 3:** Comparison of photosynthetic parameters of *C. canescens* (can), *C. hispida* (his), and *C. tomentosa* (tom) from Angersdorfer Teiche (Ang), Asche (Asc) and Bruchwiesen (Bru)

Sample	P_m_	*α*	R_d_	I_c_	I_s_
Ang_can	33.8 ± 9.6 a	0.3 ± 0.1 a	− 12.5 ± 4.5 a	40.2 ± 7.9 a	111.4 ± 23.6 a
Ang_his	42.1 ± 6.1 a	0.5 ± 0.1 a	− 21.9 ± 6.9 a	47.6 ± 1.6 ab	97.0 ± 28.8 a
Asc_tom	64.3 ± 20.2 a	0.3 ± 0.3 a	− 21.2 ± 6.9 a	98.1 ± 47.5 ab	347.0 ± 199.3 a
Bru_his	64.7 ± 18.8 a	0.2 ± 0.0 a	− 14.1 ± 1.0 a	83.3 ± 11.7 b	386.0 ± 142.6 a

Maximum photosynthesis (P_m_), dark respiration (R_d_) in mmol O_2_ h^−1^ g chl *a*^−1^ and light compensation point (I_c_), light saturation irradiance (I_s_) in µmol photons m^−2^ s^−1^ are presented. *α* in mmol O_2_ h^−1^ g chl *a*^−1^ (µmol photons m^−2^ s^−1^)^−1^ is the slope of the P/I curve at limiting irradiance. Sample size was n = 4 for the 40 min measurement of photosynthesis. Different letters indicate significant differences within photosynthetic parameters (Tukey HSD post hoc test, p < 0.05)

Photosynthesis of *C. tomentosa* from Asche was measured with the addition of CaCl_2_, KCl, MgCl_2_, NaCl and without any addition, as a control (Table 4). Differences in O_2_ evolution was found with addition of CaCl_2_, KCl and MgCl_2_, NaCl at concentrations of 2.4, 3.6, 4.8 and 6.0 mmol L^−1^. O_2_ evolution of *C. tomentosa* was higher with the addition of KCl and CaCl_2_ compared to MgCl_2_ and NaCl. At 8.4 mmol L^−1^ O_2_ evolution of *C. tomentosa* was lowest with the addition of NaCl.

**Table 4:** Difference in O_2_ evolution of *C. tomentosa* with the addition of CaCl_2_, KCl, MgCl_2_ and NaCl referred to control (without addition)

Addition	1.2	2.4	3.6	4.8	6.0	7.2	8.4
CaCl_2_	2.1 ± 2.8 a	0.1 ± 3.6 a	− 1.2 ± 3.7 a	− 1.2 ± 3.6 a	− 1.3 ± 3.5 a	− 1.0 ± 3.9 a	− 0.2 ± 3.6 a
KCl	− 1.0 ± 3.3 b	0.0 ± 4.0 a	1.4 ± 4.9 b	1.4 ± 5.2 a	1.1 ± 5.5 a	0.9 ± 5.7 ab	1.2 ± 5.9 a
MgCl_2_	− 0.6 ± 2.3 b	− 3.8 ± 2.3 b	− 4.6 ± 2.9 c	− 4.0 ± 3.6 b	− 3.4 ± 4.2 b	− 2.7 ± 4.3 bc	− 1.7 ± 4.5 a
NaCl	− 0.9 ± 3.7 b	− 5.1 ± 5.5 b	− 6.8 ± 5.4 c	− 6.2 ± 5.8 b	− 5.9 ± 5.6 b	− 5.3 ± 5.9 c	− 4.1 ± 5.6 b

1.2 mmol L^−1^ of CaCl_2_, KCl, MgCl_2_ and NaCl was added stepwise. O_2_ evolution (mmol O_2_ h^−1^ g chl *a*^−1^) is presented as accumulated concentrations (1.2, 2.4, 3.6, 4.8, 6.0, 7.2 and 8.4 mmol L^−1^). Sample size was n = 8 for the 20 min measurement of photosynthesis. Different letters indicate significant differences within a concentration level (Fischer LSD post hoc test, p < 0.05)

## Discussion

Encrustation was studied in brackish waters with strong ion anomalies. Increased ion concentrations were found in Angersdorfer Teiche, Asche and Bruchwiesen. For comparison, samples were also taken in two hard-waters lakes; Krüselinsee and Lützlower See. Carbonate precipitation was highest for charophytes in hard-water lakes and of *C. hispida* in Angersdorfer Teiche. This was not expected for *C. hispida* growing in Angersdorfer Teiche at a salinity of 6.3. Herbst et al. (2018) quantified encrustation of charophytes from freshwater and brackish water sites. Freshwater specimens had higher carbonate content of DW compared to brackish water specimens (Herbst et al. 2018). Thus, ion composition rather than ion concentration, is decisive for encrustation of charophytes. It could further explain the low carbonate precipitation of *C. tomentosa* in Asche, where Mg^2+^ exceeded Ca^2+^ concentrations, and consequently inhibits the encrustation as described by other authors (Siong and Asaeda 2009; Gomes and Asaeda 2010; Asaeda et al. 2014). Additionally, high SO_4_^2−^ concentrations in Asche and Bruchwiesen could reduce the encrustation of charophytes as Akin and Lagerwerff (1965) reported increased solubility of calcium carbonate at high SO_4_^2−^ concentrations.

The results emphasize the importance of water ion composition on carbonate precipitation of charophytes, which was strengthened regarding the site-specific pattern on encrustation along the age gradient of the plants. Here ‘age’ referred to the cell age along the plant thallus at a certain state. Site-specific patterns of encrustation were found: all parts of plant thalli (youngest, middle, and oldest) were different; the youngest differed from the other parts, and encrustation was similar for all parts of the thalli. Kawahata et al. (2013) confirmed age dependent encrustation along internodes of *C. globularis*. Younger internodes were less encrusted than older ones (Kawahata et al. 2013). Carbonate formations were also detected between the cortex and cell wall which indicated that encrustation was enclosed during forming of the cortex (Kawahata et al. 2013). Three levels of carbonate formation can therefore be distinguished; external precipitation, which can occur outside as well as intra-thallose and internal calcification of oospores which are termed gyrogonites (Raven et al. 1986; Leitch 1991; Kawahata et al. 2013).

The age pattern on encrustation in this study showed a strong site-specificity which probably arise by combination of the plant growth rate and carbonate formation rate. In both hard-water lakes, Lützlower See and Krüselinsee, age pattern on encrustation differed. The youngest parts of *C. subspinosa* and *C. tomentosa* showed less encrustation than other parts in Krüselinsee compared to individuals from Lützlower See. In Lützlower See, encrustation was similar along plant thalli. Slow growth rate and fast encrustation rate, due to higher Ca^2+^ concentration in Lützlower See, are likely to produce uniform encrustation of the whole plant.

However, in Bruchwiesen and Asche the youngest parts of plant thalli were also less encrusted than other parts, even under extreme high Ca^2+^ concentrations. Both study sites were also influenced by high concentrations of Mg^2+^ and SO_4_^2−^, which could adversely affect the carbonate formation rate. Furthermore, in the spring Bruchwiesen, temperatures did not exceed 12 °C in summer when sampling took place. Temperature, as another influencing factor, was shown to influence the precipitation of calcium carbonate (Ogata et al. 2010). Further investigations about habitat influence on encrustation of charophytes are needed to elucidate the interaction of various factors.

In Angersdorfer Teiche, the most pronounced difference in encrustation between species (*C. hispida* and *C. canescens*) and cell age (youngest, middle, and oldest part) was detected. The strong difference in species-specific encrustation could be explained by its low K/Na contents, which was 0.8 for *C. canescens* and 0.6 for *C. hispida* (Table 2). Winter and Kirst (1990) reported reduced vitality of charophytes at a ratio of K^+^/Na^+^ < 1, but found also ratios of 0.8–0.9 in *Tolypella glomerata* Leonh. 1863 and *T. nidifica* Braun 1856 (Winter et al. 1996) when exposed to a salinity of 12. Both *C. hispida*, as well as *C. canescens*, were probably growing close to their physiological limits in Angersdorfer Teiche. Photosynthesis, as a measure of the physiological state, confirmed lower P_m_ in plants (*C. canescens* and *C. hispida*) from Angersdorfer Teiche compared to those from Asche (*C. tomentosa*) and Bruchwiesen (*C. hispida*).

It is known that CO_2_ uptake for photosynthesis is connected to encrustation of charophytes (McConnaughey and Falk 1991; Raven and Beardall 2016). H^+^ pumping acidified the plant surface and increases CO_2_ concentration in the surrounding water which permeates into the cells (Beilby and Bisson 2012). The import of CO_2_ into the chloroplast generates OH^−^ which is excreted by the cell through OH^−^ channels (Beilby and Bisson 2012; Absolonova et al. 2018). Alkaline zones are formed on the plant surface where carbonate is precipitated. Beside lower photosynthesis of plants from Angersdorfer Teiche, concentration of total inorganic carbon was also lowest of investigated sites. The results imply that carbonate uptake for photosynthesis is not the only mechanism responsible for encrustation of charophytes. H^+^ is also generated by active ion transport which is linked to nutrient uptake and ion regulation (McConnaughey and Whelan 1997; Beilby and Casanova 2014). Consequently, ion regulation and osmoregulation capabilities will interfere with photosynthetic CO_2_ recruitment and it can be argued, that the low encrustation values exhibited by *C. canescens*, the only brackish water species in this study, are caused by its different osmoregulation mechanism (Winter and Kirst 1991).

Photosynthesis was affected by the addition of CaCl_2_, KCl, MgCl_2_ and NaCl. Addition of CaCl_2_, MgCl_2_ and NaCl inhibited photosynthesis; NaCl had the most adverse effect. Na^+^ toxicity is well-known; an exception is cyanobacteria which require Na^+^ for the activity of nitrogenase (Thomas and Apte 1984). Furthermore, Ca^2+^, Mg^2+^, and Na^+^ could not balance the effect of Cl^−^, whereas K^+^ compensated it. Addition of KCl enhanced photosynthesis when compared to the control measurements. It reflects the importance of ions concentration in habitats for charophyte growth.

## Conclusion

In summary, it was shown that encrustation of charophytes in water sites with strong ion anomalies could be as high as in hard-water lakes. It is assumed that ion composition, rather than ion concentration of Na^+^, Mg^2+^ and SO_4_^2−^, impact on the encrustation of charophytes. The age pattern on encrustation in this study showed a strong site-specificity, whereas encrustation of charophytes was species-specific. Encrustation, element composition and photosynthesis of charophytes were influenced by ion concentrations.

## Abbreviations

ANOVA: analysis of variance
can: *C. canescens*
DW: dry weight
his: *C. hispida*
I: irradiance
I_c_: light compensation point
I_s_: light saturation irradiance
LOI: loss of ignition
P: photosynthesis
PCA: principal component analysis
P_m_: maximum photosynthesis
R_d_: dark respiration
SD: standard deviation
sub: *C. subspinosa*
TIC: total inorganic carbon
tom: *C. tomentosa*
